# Sensory Cues Potentiate VTA Dopamine Mediated Reinforcement

**DOI:** 10.1523/ENEURO.0421-23.2024

**Published:** 2024-02-09

**Authors:** Amy R. Wolff, Benjamin T. Saunders

**Affiliations:** ^1^Department of Neuroscience, University of Minnesota, Minneapolis 55455, Minnesota; ^2^Medical Discovery Team on Addiction, University of Minnesota, Minneapolis 55455, Minnesota

**Keywords:** dopamine, optogenetics, reinforcement, value, ventral tegmental area

## Abstract

Sensory cues are critical for shaping decisions and invigorating actions during reward seeking. Dopamine neurons in the ventral tegmental area (VTA) are central in this process, supporting associative learning in Pavlovian and instrumental settings. Studies of intracranial self-stimulation (ICSS) behavior, which show that animals will work hard to receive stimulation of dopamine neurons, support the notion that dopamine transmits a reward or value signal to support learning. Recent studies have begun to question this, however, emphasizing dopamine's value-free functions, leaving its contribution to behavioral reinforcement somewhat muddled. Here, we investigated the role of sensory stimuli in dopamine-mediated reinforcement, using an optogenetic ICSS paradigm in tyrosine hydroxylase (TH)-Cre rats. We find that while VTA dopamine neuron activation in the absence of explicit external cues is sufficient to maintain robust self-stimulation, the presence of cues dramatically potentiates ICSS behavior. Our results support a framework where dopamine can have some base value as a reinforcer, but the impact of this signal is modulated heavily by the sensory learning context.

## Significance Statement

Dopamine neurons are critical for cue-based learning, and animals will work hard to activate them with brain stimulation. This self-stimulation behavior suggests dopamine transmits a reward or value signal, but recent work has emphasized dopamine's value-free functions, leaving its contribution to behavioral reinforcement somewhat unclear. We examined this question by manipulating the sensory learning context associated with dopamine neuron self-stimulation. We find that optogenetic activation of dopamine neurons in the absence of external cues is sufficient to support robust self-stimulation, but this behavior is dramatically potentiated in the presence of cues. Our results support a dual role for dopamine neurons in representing value and guiding behavior as a teaching signal.

## Introduction

Environmental cues are critical for successful decision making. Through learning, sensory events associated with rewarding experiences acquire motivational impact, gaining the power to invigorate and reinforce actions on their own. Dopamine neurons in the ventral tegmental area (VTA) are critical in this process, supporting associative learning in both Pavlovian and instrumental settings (Tsai et al., 2009; Witten et al., 2011; Steinberg et al., 2013; Hamid et al., 2016; Sharpe et al., 2017; Saunders et al., 2018; Keiflin et al., 2019).

Classic studies of intracranial self-stimulation behavior demonstrate that animals work hard to receive electrical stimulation delivered to areas of the brain associated with dopamine transmission - including the ventral midbrain, medial forebrain bundle, and striatum (Olds and Milner, 1954; Mogenson et al., 1979; Corbett and Wise, 1980; Fibiger et al., 1987; Owesson-White et al., 2008). More recently, precise cell-type specific optogenetic targeting has demonstrated that direct activation of dopamine neurons can reinforce self-stimulation behavior (Witten et al., 2011; Kim et al., 2012; Rossi et al., 2013; Ilango et al., 2014; Steinberg et al., 2014; Covey and Cheer, 2019). This supports a perspective that dopamine neuron activity can signal value in a way that mimics a natural rewarding stimulus, like food, in the context of learning and reinforcement (Wise, 2004; Berridge, 2007; Steinberg et al., 2013). Recent studies have begun to question the scope of dopamine's role in this domain, suggesting more prominent value-free functions instead, including in novelty, salience, learning rate, and movement kinematics, among other processes (Panigrahi et al., 2015; Coddington and Dudman, 2019; Engelhard et al., 2019; Hughes et al., 2020; Maes et al., 2020; Sharpe et al., 2020; Kutlu et al., 2021; Jeong et al., 2022; Millard et al., 2022; Coddington et al., 2023; Markowitz et al., 2023).

While these various interpretations are not mutually exclusive, it remains somewhat unclear to what extent brief, phasic dopamine neuron activation is itself a direct reinforcer, or if an external sensory element or salient state change is needed for a clear demonstration of ICSS behavior. Determining this is made complicated by the fact that the majority of ICSS studies either include a cue or some other reward paired with stimulation (or fail to report if cues were used), apply ICSS tests after some other learning has occurred, and/or use a targeting method that is not specific to dopamine neurons. Here, we investigated the role of sensory stimuli in dopamine-mediated reinforcement, using an optogenetic ICSS paradigm in naive TH-cre rats (Witten et al., 2011). We found that while brief VTA dopamine neuron activation (1 s at 20 Hz) in the absence of any explicit external cueing stimulus was sufficient to clearly maintain self-stimulation, the presence of cues paired with dopamine activation dramatically potentiated ICSS behavior. Our results support a framework where dopamine has some base value as a reinforcer, but the impact of this signal is flexible and scalable depending on the sensory learning context. In real-world behaving agents, where dopamine neurons are not activated in isolation during learning, dopamine's role is likely often to assign, track, and update meaning to associated stimuli and states.

## Materials and Methods

### Subjects

Male and female Th-Cre transgenic rats (*N* = 22; 15M, 7F), bred on a Long Evans background, were used in these studies. These rats express Cre recombinase under the control of the tyrosine hydroxylase (TH) promoter (Witten et al., 2011). Wild-type littermates (Th-Cre) were used as controls. After surgery rats were individually housed with ad libitum access to food and water on a 0700–1,900 light/dark cycle (lights on at 0700). All rats weighed >250 g at the time of surgery and were 5–9 months old at the time of experimentation. Rats were handled by the experimenter several times before and after surgery. Experimental procedures were approved by the Institutional Animal Care and Use Committees at the University of California, San Francisco, and at Johns Hopkins University and were carried out in accordance with the guidelines on animal care and use of the National Institutes of Health of the United States. Upon initial investigation, we found no evidence of sex-biased effects, so males and females were collapsed within each ICSS group for all reported data.

### Surgical procedures

Viral infusions and optic fiber implants were carried out as previously described. Rats were anesthetized with 5% isoflurane and placed in a stereotaxic frame, after which anesthesia was maintained at 1–3%. Rats were administered saline, carprofen anesthetic, and cefazolin antibiotic subcutaneously. The top of the skull was exposed, and holes were made for viral infusion needles, optic fiber implants, and four to five skull screws. A Cre-dependent virus coding for ChR2 (AAV5-Ef1α-DIO-ChR2-eYFP, titer 1.5–4 × 10^12^ particles/ml, University of North Carolina) was infused unilaterally (1 μl at each target site) at the following coordinates from Bregma for targeting VTA cell bodies: posterior −6.2 and −5.4 mm, lateral +0.7, and ventral −8.4 and −7.4. Viral injections were made using a microsyringe pump at a rate of 0.1 μl/min. Injectors were left in place for 5 min, then raised 200 microns dorsal to the injection site, left in place for another 10 min, and then removed slowly. Custom-made optic fiber implants (300 micron glass diameter) were inserted unilaterally just above and between viral injection sites (posterior −5.8 mm, lateral +0.7, ventral −7.5). Implants were secured to the skull with dental acrylic applied around skull screws and the base of the ferrule(s) containing the optic fiber. Headcaps were painted black, to block light transmission during laser delivery. At the end of all surgeries, topical anesthetic and antibiotic ointment was applied to the surgical site, rats were removed to a heating pad and monitored until they were ambulatory. Rats were monitored daily for 1 week following surgery. Optogenetic manipulations commenced at least 4 weeks after surgery.

### Optogenetic stimulation

We used 473 nm lasers (OptoEngine), adjusted to produce output during individual 5 ms light pulses during experiments of ∼2 mW/mm^2^ at the tip of the intracranial fiber. Light power was measured before and after every behavioral session to ensure that all equipment was functioning properly. For all optogenetic studies, optic tethers connecting rats to the rotary joint were sheathed in a lightweight armored jacket to prevent cable breakage and block visible light transmission.

### Intracranial self-stimulation training

Following recovery from surgery and at least 4 weeks for viral expression, rats were brought to the testing chambers (Med Associates) and connected to an optic cable tether to receive optogenetic stimulation. For all ICSS sessions for all groups, chamber lights were constantly illuminated, to further occlude unwanted light pollution from the laser delivery events. Two nose poke ports were positioned on one side of the chamber. For rats in the Cre+ cued and Cre− cued groups, pokes in the active port resulted in a 1 s laser train (20 Hz, 20 5 ms pulses, fixed-ratio 1 schedule with a 1 s timeout during each train), that coincided with the delivery of a cue complex – lights in the nose poke illuminated and a tone played. For rats in the Cre+ no cue group, active nose pokes produced the same 1 s laser train but with no cues. Inactive nose pokes were recorded but had no consequences. Laser stimulation parameters were chosen to align with those commonly used in other dopamine ICSS studies (Witten et al., 2011; Ilango et al., 2014; Saunders et al., 2018; Covey and Cheer, 2019; Fraser et al., 2023). This level of stimulation should evoke dopamine levels in the striatum that are comparable in magnitude and duration to a natural reward, such as sucrose (Saunders et al., 2018; Covey and Cheer, 2019; Hollon et al., 2021; van Elzelingen et al., 2022). In the no cue group, we eliminated all explicit, task-related cues that could be associated with stimulation behavior, but it remains possible that sensorimotor or interoceptive signals associated with movement and nose-poking behavior could provide some cues impacting learning.

### Extinction

The day after the final ICSS training session, rats were returned to the testing chambers for the first day of extinction. During this 1 h session, rats were tethered and nose pokes were measured as before, but laser was not delivered. For rats in all groups, no cue was presented following active nose pokes. Extinction continued for five daily sessions.

### Histology

Rats were deeply anesthetized with sodium pentobarbital and transcardially perfused with cold phosphate buffered saline followed by 4% paraformaldehyde. Brains were removed and postfixed in 4% paraformaldehyde for ∼24 h, then cryoprotected in a 25% sucrose solution for at least 48 h. Sections were cut at 50 microns on a cryostat (Leica Microsystems). To confirm viral expression and optic fiber placements, brain sections containing the midbrain were mounted on microscope slides and coverslipped with Vectashield containing DAPI counterstain. Fluorescence from ChR2-eYFP as well as optic fiber location was then visualized. A tissue from wild-type animals was examined for lack of viral expression and optic fiber placements. To verify localization of viral expression in dopamine neurons, we performed immunohistochemistry for tyrosine hydroxylase and GFP. Sections were washed in PBS and incubated with bovine serum albumin (BSA) and Triton X-100 (each 0.2%) for 20 min. About 10% of normal donkey serum (NDS) was added for a 30 min incubation, before primary antibody incubation (mouse anti-GFP, 1:1,500, Invitrogen; rabbit anti-TH, 1:500, Fisher Scientific) overnight at 4°C in PBS with BSA and Triton X-100 (each 0.2%). Sections were then washed and incubated with 2% NDS in PBS for 10 min and secondary antibodies were added (1:200 Alexa Fluor 488 donkey anti-mouse, 594 donkey anti-rabbit or 647 chicken anti-rabbit) for 2 h at room temperature. Sections were washed two times in PBS and mounted with Vectashield containing DAPI. Brain sections were imaged with a Zeiss Axio 2 microscope.

### Statistics

Behavioral data were recorded with Med-PC software (Med Associates) and analyzed using Prism 9.0. Two-way repeated measures ANOVA was used to analyze changes in behavior among the groups across training. Unpaired *t* tests were used to compare some group averages, with a Welch's correction applied when unequal variances were observed. For cumulative responding analysis, nose poke data were put into minute-long bins. Effect sizes were not predetermined. Rats were included in optogenetic behavioral analyses if optic fiber tips were no more than ∼500 microns dorsal to the VTA. Statistical significance was set at *p* < 0.05.

## Results

### Sensory cues accelerate acquisition of dopamine neuron self-stimulation

**Figure 1.**
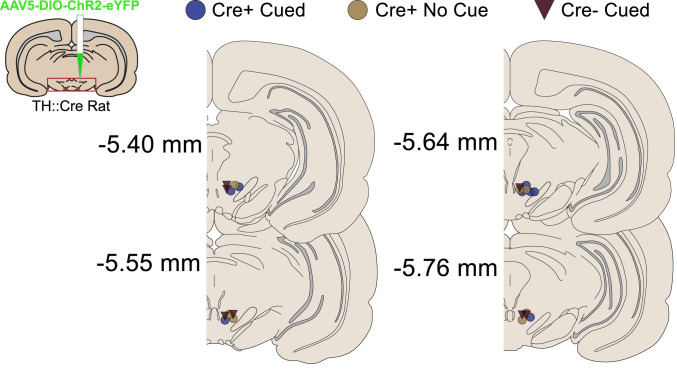
Targeting and optic fiber placements in the VTA. ChR2 was targeted to dopamine neurons in the ventral tegmental area and optic fibers were placed above the VTA for optogenetic manipulations.

**Figure 2.**
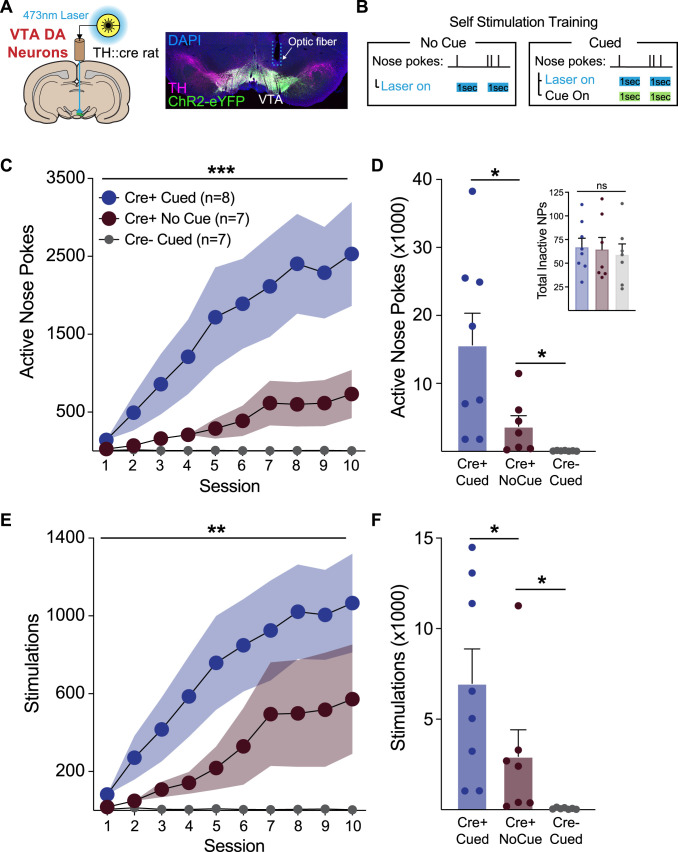
Sensory cues facilitate acquisition of VTA dopamine neuron self-stimulation behavior. ***A***, Optic fibers were targeted to the VTA for activation of ChR2-expressing dopamine neurons. ***B***, Rats were allowed to self-administer blue laser in 10 1 h intracranial self-stimulation session. Active nose pokes resulted in a 1 s blue light delivery (20 Hz, 5 ms pulses). For rats in the cued groups, this coincided with the illumination of the cue light in the nose poke port, and a delivery of a tone for 1 s. ***C***, Across training sessions, cued Cre+ ICSS rats acquired ICSS faster than no cue Cre+ rats and Cre− controls, as measured by active nose poke behavior. ***D***, Cued Cre+ rats completed significantly more active nose pokes in total, compared to the other groups. No cue Cre+ rats completed significantly more active nose pokes than Cre− controls. ***E***, Cued Cre+ acquired ICSS faster than no cue Cre+ rats and Cre− controls, as measured by stimulations received. ***F***, Cued Cre+ rats received more stimulations in total, compared to the other groups. No cue Cre+ rats received significantly more stimulations than Cre− controls. Responding among the Cre− controls remained low across all sessions. Error represents SEM. ***p < 0.001, **p < 0.01, *p < 0.05.

ChR2-YFP was targeted to dopamine neurons of the VTA in TH-Cre+/− rats (Figs. 1, 2*A*). Optic fibers were implanted unilaterally above the VTA for optogenetic excitation of dopamine neurons via blue laser light. Fiber placements were clustered over the central VTA for rats in all groups (Fig. 1).

Animals experienced 10 1 h intracranial self-stimulation sessions, during which they were tethered to an optic fiber connected to a 473-nm laser. Nose pokes into the active port resulted in a 1 s train of light delivery through the optic fiber (20 Hz, 5 ms, 2 mW pulses), which was paired with a 1 s cue (illumination of the active nose port + tone) in the Cre+ cue and Cre− cue groups (Fig. 2*B*). Cre+ no cue rats received identical laser stimulation, but with no cues. Self-stimulation behavior was substantially different across groups (Fig. 2). The presence of cues accelerated the acquisition of self-stimulation, with rats in the Cre+ cue group making more nose pokes in initial training sessions and reaching greater overall nose poke per session response levels by the end of training (Fig. 2*C*; two-way ANOVA interaction of group and session, *F*_(18,171) _= 5.92, *p* < 0.0001; main effect of group, *F*_(2,19) _= 7.24, *p* = 0.0046). Cre+ cue rats made significantly more active nose pokes overall, compared to the Cre+ no cue group (Fig. 2*D*, unpaired *t* test, *t*_(8.5) _= 2.43, *p* = 0.020). Cre+ no cue rats responded less for laser stimulation alone (Fig. 2*C*) but made significantly more active nose pokes than Cre− cue control rats (Fig. 2*D*, unpaired *t* test, *t*_(6) _= 2.35, *p* = 0.028) whose responses remained low across all sessions. Across groups, inactive nose pokes were low throughout, and there were no differences in total inactive responses (Fig. 2*D* inset, one-way ANOVA no effect of group, *F*_(2,19) _= 0.14, *p* = 0.87). Across training, Cre+ cue (*p* = 0.0039) and Cre+ no cue (*p* = 0.0078) rats, but not Cre− cue (*p* = 0.99) rats, discriminated between the active and inactive nose pokes.

We saw a similar pattern for stimulations taken. The Cre+ cue group had the most stimulations across training, finishing with a larger number of stimulations per session, compared to the Cre+ no cue group (Fig. 2*E*; two-way ANOVA interaction of group and session, *F*_(18,171) _= 3.67, *p* < 0.0001; main effect of group *F*_(2,19) _= 5.92, *p* = 0.010). Total stimulations taken were also significantly greater in the Cre+ cue versus no cue group (Fig. 2*F*, unpaired *t* test, *t*_(12) _= 2.39, *p* = 0.033). Cre+ no cue rats took significantly more stimulations than Cre− cue controls (Fig. 2*F*, unpaired *t* test, *t*_(6) _= 1.97, *p* = 0.04). Together these data indicate that dopamine neuron stimulation can support self-stimulation behavior in the absence of cues. However, acquisition of self-stimulation behavior is substantially accelerated by the presence of cues signaling dopamine neuron activation events.

**Figure 3.**
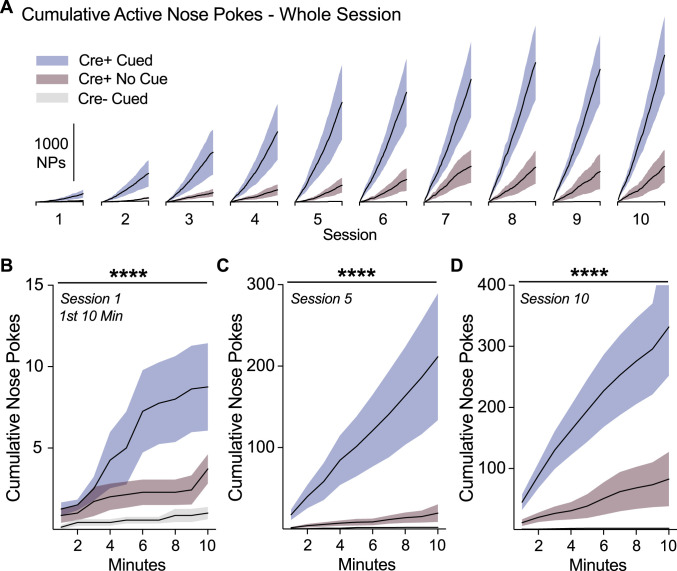
Sensory cues rapidly escalate VTA dopamine neuron self-stimulation onset. ***A***, ICSS data was expressed as cumulative active nose pokes completed within each 1 h training session, in 1 min bins. Faster responding is denoted by a steeper slope, which was evident for Cre+ cue rats versus Cre+ No cue rats. ***B***, Cumulative responding for the first 10 min of session 1 was significantly higher for Cre+ cue rats. The rapid onset of self-stimulation at the beginning of sessions continued escalate for Cre+ cue rats in session 5 (***C***) and session 10 (***D***). Error represents SEM. ****p < 0.0001.

We next examined the detailed pattern of ICSS by plotting cumulative responding within each session. The slope of response curves quickly differentiated and became super linear for Cre+ cue rats (Fig. 3), such that they responded at an accelerated pace consistently within each session, compared to no cue rats (Fig. 3*A*). The effect of cue presence on the slope of responding was apparent almost immediately in training. Cre+ cue rats were behaviorally distinguishable from Cre+ no cue rats within the first 10 min of the first training session (Fig. 3*B*; two-way ANOVA interaction of group and minute, *F*_(18,171) _= 3.86, *p* < 0.0001; main effect of group, *F*_(2,19) _= 4.71, *p* = 0.022). This early-in-session behavioral potentiation in Cre+ cue rats further escalated as training progressed (Day 5: Fig. 3*C*; two-way ANOVA interaction of group and minute, *F*_(18,171) _= 5.43, *p* < 0.0001; main effect of group, *F*_(2,19) _= 5.77, *p* = 0.011; Day 10: Fig. 3*D*; two-way ANOVA interaction of group and minute, *F*_(18,171) _= 7.55, *p* < 0.0001; main effect of group, *F*_(2,19) _= 9.83, *p* = 0.001). Together these data further support the notion that cues paired with dopamine neuron self-stimulation facilitate rapid acquisition of dopamine-seeking behavior.

### Sensory cues potentiate the vigor of dopamine neuron self-stimulation

**Figure 4.**
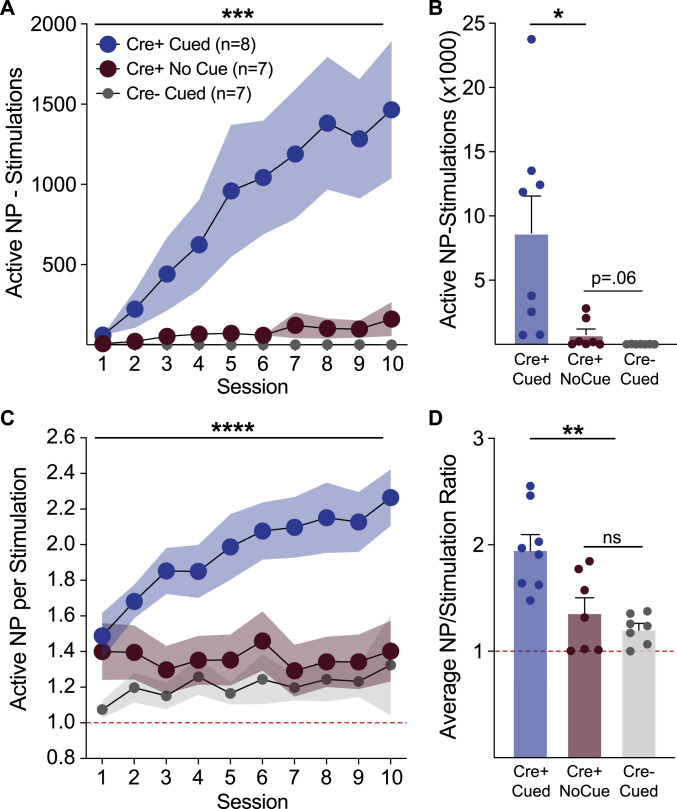
Sensory cues invigorate VTA dopamine neuron self-stimulation behavior. Cre+ cue rats exhibited more vigorous self-stimulation behavior, relative to Cre+ no cue rats and controls. ***A***, This was evident in the elevated number of active nose pokes made, compared to the number of stimulations received. Cre+ cue rats made many more active nose pokes during the 1 s timeout period that coincided with laser delivery, resulting in a positive active nose pokes stimulation value across most sessions and (***B***) in total summed across training. In contrast, Cre+ no cue rats made few extra active nose pokes during the time out period, and the sum total of extra active nose pokes was not significantly greater than Cre− controls. ***C***, Similarly, the ratio of active nose pokes to stimulations escalated in the Cre+ cue rats, relative to other groups. ***D***, The average active nose poke to stimulation ratio was significantly higher in Cre+ cue rats, and not different between Cre+ no cue and Cre− controls, despite the former showing otherwise robust self-stimulation behavior. ****p < 0.0001, ***p < 0.001, **p < 0.01, *p < 0.05.

We next quantified the rate and vigor of self-stimulation behavior, by examining the extent to which rats made “extra,” nonreinforced nose pokes. During training, in the 1 s period during which the laser was on for a stimulation event, additional active nose pokes were counted but had no additional consequence. Behavior during these “timeout” windows was significantly different across the self-stimulation groups (Fig. 4). Cre+ cue rats made many more active nose pokes during each stimulation event, as measured by subtracting the number of stimulations from the number of active nose pokes across each session (Fig. 4*A*; two-way ANOVA interaction of group and session, *F*_(18,171) _= 6.49, *p* < 0.0001; main effect of group, *F*_(2,19) _= 7.09, *p* = 0.005). Averaged across sessions, Cre+ cue rats had significantly greater total active nose pokes made during laser delivery timeouts, compared to Cre+ no cue rats (Fig. 4*B*, unpaired *t* test, *t*_(7.3) _= 2.72, *p* = 0.014). Although they made many fewer than Cre+ cue rats, there was a trend for Cre+ no cue rats to make more timeout active nose pokes compared to Cre− cue controls (Fig. 4*B*, unpaired *t* test, *t*_(6)_ = 1.7, *p* = 0.06). We also calculated a ratio score of active nose pokes to stimulations, reflecting the rate of responding per stimulation. This revealed that only Cre+ cue rats consistently showed elevated nose poke behavior above the minimum required to receive stimulations. Across training sessions the active nose poke to stimulation ratio increased for Cre+ cue rats relative to the other groups (Fig. 4*C*; two-way ANOVA interaction of group and session, *F*_(18,171) _= 2.28, *p* = 0.0033; main effect of group, *F*_(2,19) _= 11.34, *p* = 0.0006). Across all sessions, the average active nose poke to stimulation ratio was significantly higher in Cre+ cue versus no cue rats (Fig. 4*D*, unpaired *t* test, *t*_(13) _= 3.02, *p* = 0.0049). Notably, Cre+ no cue rats were not significantly different from Cre− cue controls (Fig. 4*D*, unpaired *t* test, *t*_(7.7) _= 1.04, *p* = 0.17) in average active nose poke to stimulation ratio. These data indicate that the presence of cues paired with dopamine neuron activation potentiate the intensity and vigor of self-stimulation.

### Cue history affects rate of ICSS extinction

**Figure 5.**
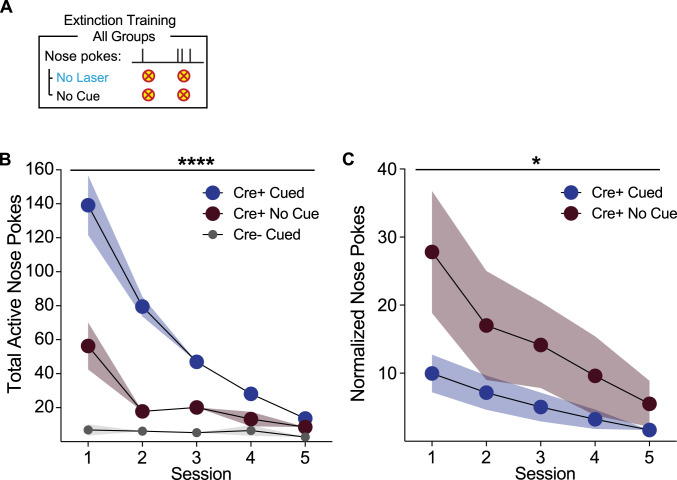
Cue history affects the rate of ICSS extinction. ***A***, Following self-stimulation sessions, rats were returned to the testing chambers and allowed to nose poke under extinction conditions, wherein no cues or laser stimulations were delivered. ***B***, Cre+ cue rats maintained higher levels of responding across extinction sessions, relative to Cre+ No cue rats and controls. ***C***, When extinction nose pokes were normalized to the final day of ICSS training, however, extinction was clearly more immediate in Cre+ cue rats, relative to Cre+ no cue rats. ****p < 0.0001, *p < 0.05.

We next examined the extinction of ICSS behavior (Fig. 5). Across five sessions, rats were again tethered to an optical cable and allowed to nose poke, where nose pokes had no consequences – no cues or laser were given (Fig. 5*A*). Behavior varied substantially across groups under these conditions. Rats trained with a cue present during ICSS training maintained a higher level of responding across extinction, compared to the other groups (Fig. 5*B*, two-way ANOVA interaction of group and session, *F*_(8,76) _= 16.18, *p* < 0.0001; main effect of group, *F*_(2,19) _= 65.85, *p* < 0.0001). However, normalizing extinction nose pokes as a percentage of the final training session nose pokes, we found that the rate of attrition of behavior in rats with a cue history was more sudden than for rats who did not have a cue during training (Fig. 5*C*, two-way ANOVA interaction of group and session, *F*_(4,52) _= 2.979, *p* = 0.027). This suggests that ICSS behavior in the Cre+ cue group had become heavily dependent on the cue's presence.

## Discussion

Here, we demonstrate that dopamine neurons can support ICSS behavior independent of explicit sensory cueing. We found that brief phasic optogenetic activation of VTA dopamine neurons in the absence of any other external signaling event was sufficient to produce reliable self-stimulation behavior compared to control animals. Critically, in the presence of explicit external cues associated with the receipt of dopamine neuron activation, we observed dramatically potentiated self-stimulation behavior. Rats experiencing dopamine stimulation paired with cues acquired self-stimulation more rapidly, took more stimulations, and responded more vigorously/at a faster rate than rats receiving optogenetic stimulation alone. Removal of cues under extinction conditions also lead to more rapid attrition of behavior in rats with a history of cue paired during training, relative to no cue rats. Our results add to a long line of research showing that electrical or optogenetic activation of dopamine neurons can support intracranial self-stimulation behavior, by clarifying that an uncued, brief increase in dopamine activity can act as a valuable, reinforcing signal akin to a natural reward.

Dopamine neurons in the VTA have long been closely linked to reward learning, motivation, and reinforcement. However, important recent work has begun to question the extent to which dopamine signals reflect behavioral reinforcement as a value signal that substitutes for natural rewards, versus a value-free teaching signal transmitting prediction, salience, novelty, internal state, exploration, or other information (Maes et al., 2020; Sharpe et al., 2020; Kutlu et al., 2021, 2022; Coddington et al., 2023; Markowitz et al., 2023). This work collectively suggests that dopamine itself, divorced from cues, state changes, movement, and other factors, carries no reward-like properties: dopamine serves as a guide of behavior, rather than reinforcing it directly. Our results support a hybrid view. First, we clarify that dopamine neuron activity can directly reinforce actions in the absence of any explicit cue-related event, which supports the classic reward value interpretation. We also show strong evidence that this value signal is malleable and can be potentiated by the presence of sensory cues that have been explicitly paired with dopamine neuron activity. This suggests that dopamine neuron signals do not necessarily represent a set static value, but the impact of a given dopamine event is modulated by other features of the learning context, including additional sensory information to support the establishment of associative contingencies. Consistent with this, previous work has shown that in the context of Pavlovian learning, noncontingent, unsignaled dopamine neuron activation does not produce obvious behavioral consequences, including nonspecific movement (Coddington and Dudman, 2018; Saunders et al., 2018; Pan et al., 2021). In contrast, if that stimulation event is signaled to the animal with a sensory cue, or if it occurs following a specific action, robust learning and behavioral reinforcement can occur.

Our results suggest that phasic dopamine transmission has the ability to instantiate some basic value to directly support reinforcement, but that signal must interact with other brain processes and states evoked by cues, rewards, and actions for its impact to be fully realized. The extent to which dopamine's contribution to the net “value” within a learning and decision-making context will likely depend on the details - the identity, duration, configuration, and learning history, etc. - of those other features. This makes sense as, in real behavioral contexts, where dopamine neurons are never activated in isolation, a myriad other processes modulate dopamine neuron activity and function, including sensory, homeostatic, ingestive, and motor control systems (Redgrave and Gurney, 2006; McCutcheon, 2015; Tian et al., 2016; Coddington and Dudman, 2019; Grove et al., 2022). Thus, value is not solely the domain of dopamine, and in many cases, the value component of dopamine's contribution to a given behavior state may be negligible (Adamantidis et al., 2011; Maes et al., 2020; Sharpe et al., 2020; Zell et al., 2020; Coddington et al., 2023; Markowitz et al., 2023). Collectively, this supports a framework where VTA dopamine serves an important role in credit assignment (*what led to that?*) as well as evaluation (*how was it?*).

Our results are consistent with a recent study (Fraser et al., 2023) demonstrating that VTA dopamine neuron activity supports flexible self-stimulation behavior. Rats will engage in complex seeking tasks, including completing a chain of actions, to receive VTA dopamine neuron activation. Further, the presence of cues paired with VTA dopamine neuron activation promotes higher breakpoints on a progressive ratio schedule. While we did not manipulate the effort required to self-stimulate, collectively our data and this study suggest that VTA dopamine neurons can assign value to stimuli and actions, which facilitates both vigorous but also flexible behavior. In contrast, self-stimulation of SNC dopamine neurons, while similar to VTA levels for continuously reinforced responding, does not persist when multiple actions are required to produce stimulation (Fraser et al., 2023). Thus while VTA and SNC dopamine neurons may broadly signal a similar base value (Ilango et al., 2014; Hollon et al., 2021), the learning processes they facilitate are qualitatively different (Wise, 2009; Howe and Dombeck, 2016; Saunders et al., 2018; Keiflin et al., 2019).

In our studies, the presence of an explicit cue dramatically elevated dopamine neuron self-stimulation behavior, where some rats made *thousands* of responses in 1 h. Cued rats not only received many more stimulations than noncued rats, they also nose poked at more than double the rate. This particular expression of self-stimulation could be consistent with the development of compulsive-like behaviors thought to underlie addiction and related diseases. While we did not assess the extent that cued ICSS was potentially more resistant to devaluation or punishment, our data suggest that cues play a prominent role in the development of highly vigorous dopamine seeking, which could be indicative of a compulsive-like phenotype. Recent studies have shown that individual differences in the extent to which dopamine neuron self-stimulation becomes compulsive-like relate to the emergence of unique plasticity within corticostriatal circuits (Pascoli et al., 2015, 2018). Notably, individual differences in cue responsivity and dopamine activation patterns likely interact to produce different vulnerabilities in these disease states (Flagel and Robinson, 2017; Poisson et al., 2021).

The conceptual landscape for VTA dopamine's functions grows ever more complex, as new tools and models allow for greater precision in manipulation and measurement of the system during behavior. Our results are consistent with an emerging view of VTA dopamine as both reward and value-free teaching signal. This work, in combination with several other recent studies, highlights the importance of the learning context in which dopamine's functions are being probed, for determination of its function.

## Synthesis

Reviewing Editor: Jibran Khokhar, Western University Department of Anatomy and Cell Biology Decisions are customarily a result of the Reviewing Editor and the peer reviewers coming together and discussing their recommendations until a consensus is reached. When revisions are invited, a fact-based synthesis statement explaining their decision and outlining what is needed to prepare a revision will be listed below. The following reviewer(s) agreed to reveal their identity: Ali Mohebi.

The reviewers agreed that this paper makes valuable contributions to our understanding of the role of dopamine in learning and reinforcement. They did, however, present some suggestions to clarify the interpretations of the findings, which can be found in their reviews below.

## Author Response

We thank the reviewers for their thoughtful and constructive feedback on our manuscript. We have revised the paper in response to all comments, including text edits, additional references, and a new data figure - edited sections in the manuscript are labeled as orange text. A detailed response and description of those changes is presented below:

Reviewer 1

Reviewer: One of the main conclusions is that "brief phasic optogenetic activation of VTA dopamine neurons in the absence of any other signaling event was sufficient to produce reliable self-stimulation behavior". I find this conclusion imprecise, as one could argue that rats in the cre+NoCue group experience a plethora of interoceptive and proprioceptive cues related to the action of poking their noses into a metal hole. When rats poke their noses, their whiskers bend, their visual landscape changes and (probably) feel the cold touch of the aluminum material of which the hole structure is likely made of.

Within this interpretation, the difference in responding between the cue+ and cue- groups could be attributed to the different cue-reward contingencies existent between interoceptive and exteroceptive cues. With the protocol used, the light and tone cue has a perfect contingency with the reward delivery. Interoceptive cues, however, have a poorer contingency, as not all nosepoke hole contacts are rewarded and many other interactions with the operant box (walls, floor, etc) elicit similar interoceptive cues that are not paired with the self-stimulation. This is an idea that could have been easily explored by iteratively reducing the contingency between the reward and the exteroceptive cues but there authors do not even mention interoceptive cues in the entire manuscript.

I understand that, in order to address my concern, one would have to get rid of such an interceptive landscape, which is virtually impossible in the current instrumental paradigm. However, authors' should provide convincing arguments as to why they think no cues at all were present in the cre+NoCue group, even when considering all the interoceptive cues potentially available to the rat.

Response: We thank the reviewer for this thoughtful comment. It is true that our studies do not rule out interoceptive cues/states as potentially contributing to the ability of dopamine neuron stimulation to reinforce behavior in the absence of any explicit external cues. Indeed, the state change of stimulation event itself - in terms of the perceptible neural and physiological impact it has - is very likely a critical component of what drives behavior in the "No cue" group in our dataset.

We have revised the paper to clarify this point - emphasizing that we mean specifically task-related external cues- and present the caveat that it is possible that some elements of external bodily feedback, including subtle sensorimotor factors, could play a "cueing" role that could promote ICSS in the groups that do not have a light and tone paired with laser delivery. The discussion section also includes reference to previous studies wherein dopamine neuron optogenetic activation, when delivered unpaired/noncontingent with cues and actions, does not produce obvious behaviors. This suggests that some state change, either interoceptive or exteroceptive, is an important part of determining if dopamine neuron activity has an enduring impact on behavior. Removing literally all states and stimuli would effectively eliminate the instrumental nature of the behavior, and change the overall question, but that would certainly be an interesting future direction.

Reviewer: Dopamine release effects of TH+ neuron optostimulation are extremely sensitive to stimulation parameters. Authors argue that their findings refine our view of VTA dopamine function in learning -in general-, but it is unclear whether their optostimulation parameters yield naturally-occurring levels of dopamine terminal release in learning contexts. Can the authors provide supplementary/representative data supporting that their stimulation resulted in physiological levels of dopamine release (for instance in the NAc)? If they do not have this data at hand, citing previously published research using similar stimulation parameters would be enough.

Response: This is indeed an important point, one we specifically considered in selecting our stimulation parameters. We selected a brief stimulation event (1 second) to ensure that a physiological range of dopamine levels were evoked and for consistency with the range of stimulation durations that are common in the ICSS literature, which is important for comparing out results to previous work. Based on a number of previous studies (PMID: 34637751, PMID: 30970251, PMID: 35594399) as well as a broad review of the literature of dopamine recordings, 20hz 1-second optogenetic activation should produce dopamine release in the nucleus accumbens that is in the general range of duration and similar in magnitude to that produced by a natural reward, such as sucrose consumption. In some studies (PMID: 30038277, Suppl Figure 5), it has been shown that even longer optogenetic stimulation events (up to 5 seconds) can evoke dopamine neuron signals that are comparable to those produced by large sucrose reward deliveries. We have added additional references to the manuscript to emphasize these points.

One conclusion from comparing stimulation vs reward-evoked dopamine signals across various studies is that, just as different stimulation types produce variable dopamine, so do different rewards (modalities, sizes, preferences, modes of consumption, etc), and so there is no single "natural" or "physiological" dopamine level. While no electrical or optogenetic stimulation is perfectly "physiological" in terms of the coordinated activity produced, the broad similarity with which optogenetic and natural rewards can shape learning, in our studies and others using optogenetic manipulations of dopamine neurons, also suggests that the manipulations fall within the range of neural changes that the brain can create on its own.

Also relevant to the discussion of compulsive dopamine neuron ICSS, it remains possible that certain types of optogenetic stimulation may over-drive dopamine circuits and produce plasticity that would not be seen with normal rewards (but could be relevant to drug exposure, for example). Given the very brief stimulation in our dataset, we do not think that the type of learning engaged is qualitatively different from that engaged by a palatable natural reward.

Reviewer: At the end of the discussion, it is concluded that the presented 'data suggest that cues play a prominent role in the development of compulsive dopamine seeking'. The study does not perform any experimental assessment of compulsive-like behaviors. I assume the authors interpret the increase in nosepoke/stimulation ratio in the cued group as a compulsive-like phenotype, but this is not openly stated or clearly justified. I understand this is a common interpretation within the field of self-administration studies, but additional phenotyping efforts should be performed in the same rats in order to relate this increase to a greater compulsive phenotype.

Response: We apologize for the lack of clarity in this section of text. In the discussion, after describing our results, we attempt to connect them to the broader dopamine literature and discuss other relevant studies. In the referred paragraph, we highlight some recent work showing that dopamine neuron self-stimulation can become "compulsive" in that is it resistant to punishment, and simply make the point that cues may play a role in such a process, which the authors of those studies also argue. Based on the current studies, we remain agnostic to whether or not vigorous cued self stimulation represents compulsive behavior in our hands. It is definitely possible that the heavily increased response rate in the cue+ group could be related to compulsive behavior, but that would need to be tested in a future experiment. The discussion has been revised to clarify this point.

Reviewer: Why are individual differences mentioned in the discussion? This paper, despite having the data available, does not conduct any kind of individual differences-based analysis.

Response: Similar to the previous comment, at this stage of the discussion section - which is meant to connect these results to the larger literature - we comment on how individual differences may be an important additional factor in determining the relationship between cues and dopamine-mediated reinforcement, since there is well documented variability in rats' responsivity and valuation of cues, including in the compulsive dopamine ICSS studies mentioned above. We apologize for the lack of clarity in this section and have revised the discussion to clarify that we are not studying individual differences in this specific dataset.

Reviewer: The authors state that the vigor of self-stimulation behavior was examined by determining the extent to which rats made 'extra' nose pokes. However, the relationship between these two elements is not explained and it is not clear to me. Could the authors justify how is this relationship conceptually defined?

Response: Thank you for this comment, which is useful for a general revision to the paper to clarify the notion of vigor (vs effort, etc). In this case, "extra" nose pokes are those active nose pokes that are technically not reinforced - occurring during the 1-sec long stimulation window, vs the active nose pokes that specifically led to each laser delivery. Cue+ rats made many more extra nose pokes in the active port, which also means they responded faster/more vigorously compared to the no cue rats. Cre+ cued rats, by the end of ICSS training, are responding at about three times the rate of the Cre+ no cue rats. The speed/frequency/intensity of behavior is a central component of "vigor", and we therefore feel that use of this term is justified. In the revised paper, we have included a new figure (New figure 3) featuring cumulative responding data for ICSS behavior. This further shows that the presence of cues facilitates the acquisition of vigorous ICSS. The new analysis indicates this effect is rapid - apparent within the first few minutes of session 1, and that cues result in a faster escalation of ICSS within sessions across training. This further supports the notion that cues invigorate ICSS, in that behavior occurs at a faster rate. Examining within session data also demonstrates that Cue+ rats maintain a steady elevated rate of ICSS, rather than occurring in a burst-pause fashion. We think this new analysis offers more clarity and insight for the manuscript's conclusions.

As pointed out by the other reviewer, the distinction between vigor and effort was not particularly clear. We have clarified this distinction in the revised manuscript as well - noting specifically that we have not manipulated the effort required to self-stimulate.

Reviewer: The Mann-Whitney test is a nonparametric method commonly used to compare ranks. However, the authors use this statistic for most of their comparisons, which to me seem parametric. If violations of homoscedasticity or normality are detected, Geisser-Greenhouse corrections, or similar, should be used to correct F statistics (ANOVA).

Response: We had originally included Wilcoxon tests just for the group average comparisons (ANOVAs are used for all the main group by session data, with appropriate corrections if needed) because of unequal variances among the group summed data in some cases - this is a common approach for this kind of data (PMID: 37185097). Nevertheless, per the reviewer's suggestion in the revision we have rerun those comparisons with a parametric test including a Welch's correction to account for unequal variances when appropriate, and updated the manuscript accordingly. This had no effect on the overall data conclusions.

Reviewer 2

This manuscript confirms the reinforcing effects of dopamine on specific actions using optogenetic stimulation. Furthermore, they present a novel finding that pairing this stimulation with an external cue enhances this reinforcement. While the former is consistent with prior research, the latter represents a noteworthy observation that advances our understanding of dopamine's cue-mediated reinforcing effects. Given these contributions, I support the publication of this paper in eNeuro. However, I remain somewhat uncertain about the interpretation of the results, and I would like to ask the authors to modify their text. I have listed a few points below:

Reviewer: The authors employ the number of nose pokes during the 1s stimulation period as a measure of vigor, but a more robust justification for this choice is needed. Vigor is commonly assessed through parameters like task engagement latency and the exerted effort to complete a task. It remains unclear how accurately the selected measure reflects vigor, as it appears to lean more towards indicating compulsive behavior.

Response: Thank you for this comment. We agree that there are other behavioral factors that may relate to the notion of vigor but in our interpretation here, we simply mean it in the most basic sense, related to the intensity/frequency/speed of behavior. Rats in the cued group engage in nose poking at a much faster rate (ie. more vigorously) than no cue rats - about 3x faster by the end of training - which supports the conclusion that ICSS is more vigorous in general in the cued group. We have avoided the term "effort" in the manuscript as we are not explicitly manipulating effort required to self-stimulate. Although we would argue that it is more effortful to nose poke fast versus slow, and so the cued rats are to an extent engaging in more effortful behavior. In the revision we have tried to clarify this point in terms of what we mean by vigor. We agree that the vigorous behavior in the cued group could be indicative of a compulsive-like phenotype, but this suggestion is made with caution as we have not directly assessed the resistance of ICSS to devaluation/punishment.

As described above, we have included a new figure of cumulative responding data for ICSS, which shows that Cue+ rats rapidly escalate the rate of their self-stimulation, even within the first minutes of each session, compared to no cue rats. We think this further supports the notion of elevated vigor of responding in the cue+ group, in addition to adding further insight into the pattern of behavior for self-stimulation.

Reviewer: In the discussion, the authors suggest that dopamine neuron activity can directly reinforce actions in the absence of any other sensory or cue-related event, which supports the classic reward value interpretation.' However, I find it challenging to connect how the reinforcing action of dopamine supports the traditional, albeit refuted, notion of dopamine functioning as a reward/pleasure signal. The paper's title suggests that the authors view dopamine as a reinforcing signal, not a reward signal. This distinction is not clearly communicated in the text, which could potentially mislead many readers.

Response: Thank you for this thoughtful comment. As a point of clarification, by "reinforce actions" we simply mean that dopamine stimulation promotes the acquisition and maintenance of operant behavior. The term reward is indeed more general, and in the original manuscript we more or less use it synonymously with reinforcement in that - a reward is reinforcing, a conclusion that the ICSS behavior in the no cue group adds further support to. In our (and we think many others) view, dopamine is a reward, in that is it valuable and supports learning and reinforcement. The notion of pleasure is interesting as dopamine's direct role in pleasure, which can be a component of reward but is a distinct process, as the reviewer points out, has been generally refuted - we make no claims of pleasure or palatability related roles for dopamine in these studies.

Reviewer: The paper frequently characterizes dopamine as a value-free signal. However, it's important to note that the current experiments do not involve direct manipulation of value. Therefore, the results may not decisively contribute to this ongoing debate within the dopamine research community.

Response: As a point of clarification, in the manuscript we attempt to connect our results to the larger dopamine literature, where a number of recent studies have argued that dopamine signals within reward-learning contexts are not conveying value (at all), but merely teaching. That is not something that we are explicitly arguing here and our studies are not specifically set up to test that. Our results do show that dopamine stimulation does convey some value, in that it supports reinforcement in the absence of explicit external cues - the dopamine stimulation is valuable in that it is sought out.

We've revised the discussion overall to help clarify these points. We do believe that it is entirely appropriate to, in a discussion section, cover relevant literature and to connect different features of data in service of contributing to the field. We also do not assert that these results decisively conclude ongoing debate about dopamine functions.

Reviewer: The prevailing model of dopamine function posits that dopamine signals a reward prediction error, utilized by downstream circuits to reinforce actions, among other functions. While this view has recently been challenged, the paper's concluding statements make assertions that extend beyond the demonstrated results, such as "Our results support a view of VTA dopamine as both reward and value-free teaching signal."

Response: We appreciate this comment. This statement, in the context of discussion of the broader literature of dopamine function, was meant to indicate that our results are generally supportive of a view that incorporates value and value-free learning information in dopamine neurons. We've revised this section to clarify that the current experiments do not specifically test that but are merely broadly consistent with that interpretation.

Reviewer: I suggest including the following papers in the bibliography:

- Hollon, Nick G., et al. "Nigrostriatal dopamine signals sequence-specific action-outcome prediction errors." *Current Biology* 31.23 (2021): 5350-5363.

- Zell, V., Steinkellner, T., Hollon, N. G., Warlow, S. M., Souter, E., Faget, L., ... & Hnasko, T. S. (2020). VTA glutamate neuron activity drives positive reinforcement absent dopamine co-release. *Neuron*, *107*(5), 864-873.

- Hamid, Arif A., Jeffrey R. Pettibone, Omar S. Mabrouk, Vaughn L. Hetrick, Robert Schmidt, Caitlin M. Vander Weele, Robert T. Kennedy, Brandon J. Aragona, and Joshua D. Berke. "Mesolimbic dopamine signals the value of work." *Nature neuroscience* 19, no. 1 (2016): 117-126.

- Covey, Dan P., and Joseph F. Cheer. "Accumbal dopamine release tracks the expectation of dopamine neuron-mediated reinforcement." *Cell reports* 27, no. 2 (2019): 481-490.

Response: Thank you for the suggestions. These references have been added to the revision.

- The analysis for Fig.4 may benefit from further clarification. Consider presenting the number of active nose pokes normalized by their value in the session immediately preceding extinction. Alternatively, showing the rate/slope of extinction could provide a clearer depiction of the results.

Response: We apologize for the lack of clarity in this figure. Panel 5C (formerly Figure 4C) shows one version of what the reviewer suggests - active nose pokes normalized (in this case as a percentage) to the nose pokes that occurred in the training session before extinction. We have clarified the text to denote this normalization and what it references. This analysis shows that removal of the cue during extinction facilitates faster cessation in responding in the Cue Cre+ group, which generally supports the notion that in those animals responding at a high level was critically dependent on the presence of the cue.
